# The Factors Associated with Access to Healthcare Services for Cancer Patients in Saudi Arabia

**DOI:** 10.3390/healthcare14101399

**Published:** 2026-05-20

**Authors:** Zahraa Alakrawi, Nouf Al-Kahtani, Alaa Alsaffar, Bayan Alhamadah, Nada Altawal, Hassan Aljumaia, Kawther Alakrawi, Hayat Mushcab

**Affiliations:** 1 Department of Health Information Management and Technology, College of Public Health, Imam Abdulrahman Bin Faisal University, P.O. Box 1982, Dammam 31441, Saudi Arabia; zalakrawi@iau.edu.sa (Z.A.); nkalkahtani@iau.edu.sa (N.A.-K.); alaa10ali10@gmail.com (A.A.); bayan.alhamadah@gmail.com (B.A.); altawalnada.01@gmail.com (N.A.); 2 Ministry of Health, P.O. Box 2477, Al Ahsa 31982, Saudi Arabia; hassan5211@gmail.com; 3 King Abdulaziz University Dental Hospital, Jeddah 21589, Saudi Arabia; 4 Research Office, Johns Hopkins Aramco Healthcare, Dhahran 34465, Saudi Arabia

**Keywords:** risk factors, access to healthcare services, cancer, disease progression, virtual appointment, health belief model

## Abstract

Background: Cancer is a chronic disease with significant health impacts and is a leading cause of mortality worldwide. Cancer patients often require frequent hospital visits to manage their condition effectively. Therefore, understanding the factors that influence their ability to access healthcare services—such as age, gender, citizenship, region of residence, educational level, and income—is crucial, as these factors can impact continuity of care and overall quality of life. Purpose: This study aims to identify the factors determining cancer patients’ healthcare access and to propose alternative solutions that will enhance their ability to access services. Methods: This cross-sectional quantitative study utilized the health belief model for data analysis. Data were collected randomly through an online questionnaire targeting cancer patients across Saudi Arabia. Results: The findings indicated that payment method, distance to healthcare facilities, tumor type, and willingness to use virtual appointments were significantly associated with access to healthcare services. A total of 391 participants were included, the majority of whom were female (*n* = 291), aged 39 to 48 (*n* = 111), Saudi citizens (*n* = 376), residing in the Eastern region (*n* = 210), holding a bachelor’s degree (*n* = 193), and reporting no monthly income (*n* = 110). Conclusions: Access to healthcare services for cancer patients in Saudi Arabia is challenged by several factors, including payment methods, travel distance, cancer type, and the acceptance of health applications. Promoting digital health tools and virtual appointments can significantly improve access to care and facilitate ongoing management for cancer patients.

## 1. Introduction

Cancer is among the top ten causes of death worldwide [1,2]. In 2021, the World Health Organization recorded an increase in the number of deaths resulting from lung, trachea, and bronchial cancers, from 1.2 million in 2000 to 1.9 million in 2021, making these conditions the sixth most common cause of death [2]. Cancer patients require consistent access to healthcare facilities for essential activities such as screening, treatment, and follow-up care; however, numerous barriers hinder their access to these services [3]. Evidence suggests a strong link between obstacles to healthcare access and the progression of cancer, indicating that these barriers can significantly affect the progression of a patient’s disease [3].

Regular follow-up is crucial for cancer patients, yet many face challenges in reaching healthcare facilities due to a range of factors, including geographic limitations like distance, as well as economic constraints [4,5,6]. In developing countries, access to healthcare is often severely restricted, with cost being the primary barrier, resulting in many individual patients facing inadequate health service access [7,8]. Research indicates that people in less developed countries experience greater difficulties accessing healthcare than those in wealthier countries. Limited financial resources can create significant obstacles to obtaining necessary care, and the relationship between access to healthcare services and low income is mutually reinforcing—delayed or unmet healthcare needs can lead to deteriorating health, resulting in lost income and, in turn, increased healthcare expenses [6,9,10,11].

In addition, patients often encounter challenges related to transportation, particularly those undergoing treatments such as radiation therapy. Rising gasoline and parking costs can exacerbate these difficulties, particularly for patients in rural areas who are especially vulnerable to fluctuations in fuel prices [12]. Consequently, distance from the relevant facility is considered a critical factor affecting access to healthcare—the likelihood of utilizing needed services decreases as the distance between a patient’s residence and healthcare facilities increases. Geographic distance poses a real challenge, as it often necessitates extensive travel to receive care [4,13,14,15]. Transportation issues further complicate this barrier, making it harder for patients to reach healthcare providers [16,17]. Another crucial determinant is level of education; individuals in rural areas with lower educational attainment are more likely to rely on traditional medicine, whereas those with higher education tend to seek regular medical examinations [18,19]. Additionally, people with lower educational levels face language and cultural barriers that can affect access to healthcare services [18,19].

On the other hand, several methods have been shown to significantly improve access to healthcare services and have come to be considered effective solutions. One notable approach is the use of Patient Navigation Programs (PNPs), which aim to enhance cancer care delivery by identifying and addressing the barriers that restrict access to quality medical care. Patient navigators serve as valuable resources for both patients and providers, assisting throughout the entire continuum of care, including primary prevention, screening, treatment, and survivorship support [20,21].

Additionally, modern healthcare delivery methods, such as virtual appointments, leverage remote technology to help cancer patients overcome geographical barriers, an approach that minimizes unnecessary travel for care. A study by Fortney et al. (2011) highlights various electronic health technologies that facilitate virtual communication between patients and healthcare providers beyond traditional face-to-face encounters [18,22]. Current digital communication tools include interactive video consultations, personal monitoring devices, and personal health records [18]. Enhancements in digital access to healthcare services could significantly alleviate the geographical and temporal barriers many patients face [23]. Moreover, the health sector transformation program under the Kingdom’s Vision 2030 focuses on public health in all its aspects, aiming to facilitate access and improve the quality of healthcare services. This will be achieved through the development and enhancement of hospitals, healthcare centers, and emergency services, as well as the promotion of digital transformation in healthcare. The program emphasizes prevention over treatment, maintaining human health, and improving road safety. In alignment with the goals of Vision 2030, to enhance the quality of life and improve the efficiency of healthcare services, it aims to improve cancer patient care within a “modern care model” by promoting early detection, providing advanced treatments, and offering psychological support [24].

The Health Belief Model (HBM) is one of the most widely used theoretical frameworks for explaining health-related behaviors and healthcare utilization. Originally developed by Rosenstock and later expanded by Becker and colleagues, the HBM proposes that individuals’ engagement with health services is influenced by several key constructs: perceived susceptibility, perceived severity, perceived benefits, perceived barriers, cues to action, and self-efficacy [25,26,27,28]. The model suggests that individuals are more likely to seek healthcare when they perceive themselves to be at risk of a serious condition, believe that the benefits of action outweigh the barriers, and feel confident in their ability to access and use healthcare services.

In the context of cancer care, the HBM is particularly relevant because cancer management requires continuous engagement with healthcare systems for screening, treatment, and follow-up. Previous studies have demonstrated that perceived barriers such as financial burden, travel distance, and system complexity significantly influence cancer patients’ healthcare utilization [27,28]. Additionally, digital health tools and virtual appointments may function as cues to action and facilitators that enhance perceived benefits while reducing access barriers. Therefore, applying the HBM in the present study provides a structured framework to understand how individual perceptions and structural factors jointly influence access to healthcare services among cancer patients in Saudi Arabia.

This study explores the factors that affect access to healthcare services for cancer patients in Saudi Arabia. Because continuous follow-up and frequent medical appointments are crucial for these patients, barriers to access can lead to neglected treatment and worsening health; therefore, it is essential to identify these factors and amplify the voices of patients facing access challenges. The factors examined in order to propose solutions that will improve access to care include age, gender, citizenship, region of residence, education level, income, type of tumor, payment method, distance, and technology used. While similar studies have been conducted in other countries, this research fills a gap in Saudi Arabia, providing valuable insights for the Ministry of Health, cancer patients, and the broader community.

## 2. Research Methodology

### 2.1. Research Design

This study employed a cross-sectional design that encompassed the period from 21 March 2023 to 21 March 2024. We invited diverse populations across Saudi Arabia to complete an online survey. The data collection instrument used in this study was a structured, autonomous online questionnaire designed based on an extensive review of the literature and adapted from previous research on identical topics [1,5,6,10,13,15,17,29,30,31]. It has been revised and reorganized to better suit our research and its needs, guided by the Health Belief Model framework. The questionnaire was meant to investigate factors associated with access to healthcare services for cancer patients in Saudi Arabia. The questionnaire was created and distributed using the QuestionPro (QuestionPro Inc., Austin, TX, USA), a web-based survey platform, and consisted of two main sections. Section A: Sociodemographic characteristics included 8 items assessing participants’ age, gender, nationality, region of residence, educational level, and monthly income. Section B: Access to healthcare services dimensions included 18 items measuring healthcare access-related factors such as type of tumor, type of cancer, reason for last hospital visit, distance to healthcare facilities, method of payment, financial support, type of cost, use of virtual appointments, and acceptance of health applications. Certain items utilized closed-ended formats and were assessed through binary responses (Yes/No) or multiple-choice alternatives. The approval of health applications was evaluated with a 5-point Likert scale that ranged from strongly agree (5) to strongly disagree (1). To quantify overall accessibility, an access to healthcare score was calculated by summing selected binary items, where Yes = 1 and No = 0. Higher scores indicated better access to healthcare services. The full questionnaire is provided in Appendix A: The Data Collection Instrument.

### 2.2. Ethics and Limitations

The study was approved by the Institutional Review Board (IRB) at Imam Abdulrahman bin Faisal University, Saudi Arabia, on 21 March 2023; the ethical approval number is IRB-UGS-2023-03-133. This study was limited to a sample of cancer patients in the Saudi population, and other samples in different cultural and socioeconomic settings may yield different findings. A signed consent form and approval to publish the results were obtained from the participants through the online survey itself.

### 2.3. Study Setting

The online survey was created using the QuestionPro platform and distributed via social media platforms such as WhatsApp (through shared links in groups and direct messages), Telegram, Instagram, and TikTok across all regions of Saudi Arabia.

### 2.4. Participants

The study participants were recruited using the purposive sampling design. The sample size for this study was determined using the Epi Info software tool, which is widely recognized in epidemiological statistics. Based on the latest statistics from the Saudi Cancer Registry, the population of interest consists of 16,859 cancer patients, representing the total number of cancer cases in the Kingdom as of 2016. Consequently, the minimum required sample size was calculated to be 376 cancer patients. A confidence level of 95%, a margin of error of 5%, and an anticipated response rate of 50% were established.

The study included citizens and residents of Saudi Arabia across all categories of gender, age, income, and other demographics; however, individuals under 18 and those diagnosed with benign tumors were excluded from participation.

### 2.5. Data Collection

#### 2.5.1. Research Instrument

The primary instrument for data collection in this study was an online survey. The survey questions were developed based on the existing literature and aligned with the study’s primary objective of efficiently gathering information on the characteristics of a diverse sample population. The Appendix A provides further details on the research instrument.

The survey was administered using the QuestionPro platform, through which the questionnaire was created and disseminated to the Saudi community. To maximize reach, the survey was shared via various social media applications, including WhatsApp and Telegram.

Five independent variables were analyzed in order to investigate the factors associated with access to healthcare services: age, gender, geographical location, economic conditions, and educational attainment. Access to healthcare services was designated as the dependent variable. Excel and SPSS were utilized for data manipulation and analysis.

#### 2.5.2. Validation Process

As the questionnaire was intended for distribution to patients who use Arabic as their first language, there was a need to translate it from English to Arabic. Two experts in the English and Arabic languages did the translation. The translation process consisted of two steps. The first step was translating the original English questionnaire into the Arabic language by one of the experts. The new Arabic version was then sent to the second expert via email to translate it back to English to validate the translation and ensure that the language was correct and understandable. Independent reviews of the original and the re-translated version were performed to ensure the accuracy of the wording.

#### 2.5.3. Content Validity

The content validity of the questionnaire was assessed by eight experts who were considered acceptable for a content validity assessment. The participating experts were from both academic and healthcare backgrounds. The validation instrument consisted of two main sections. The first part contains questions for the expert regarding the relevance of the questions in the questionnaire to the study purpose, the clarity of the questions, and the ease of responding. The second part contained an open-ended question requesting the expert’s comments on the tool, the sequence of the questions, the length of the tool, and any other comments on how to enhance the benefits of the tool. The data collection instrument was modified based on the experts’ input and comments.

### 2.6. Statistical Analysis

All statistical analyses were conducted using the Statistical Package for Social Sciences (SPSS). A combination of independent sample tests and ANOVA was employed to evaluate the significance of associations between the various factors—namely age, gender, citizenship, region, educational level, monthly income, type of payment, distance, type of cost, financial support, type of financial support, type of tumor, type of cancer, reason for the last visit, virtual appointment usage, and acceptance of virtual appointments.

The responses to these questions, which reflect patients’ ability to access healthcare services, were utilized to construct an access scale. For example, participants were asked, “Have you ever missed an appointment due to expense?” with responses coded as follows: Yes = 1 and No = 0. To quantify overall accessibility, the coded responses from each question were aggregated to yield a final score.

Statistical significance was determined at a *p*-value of less than 0.05. Notably, factors such as type of payment, distance, type of tumor, and acceptance of virtual appointments demonstrated statistical significance, while the remaining factors did not meet this threshold.

Also, to examine the dimensional structure of the composite access score, tetrachoric correlations were computed among the seven binary access items, and three sub-domain scores were derived based on the resulting cluster structure: geographical (3 items), financial (2 items), and digital (2 items). Differences in sub-domain scores across relevant predictors were assessed using one-way ANOVA. Additionally, an exploratory multivariable linear regression was conducted to assess the stability of the bivariate associations, with the composite access score as the outcome and payment type, distance, and health application acceptance entered simultaneously as predictors. Statistical significance was set at *p* < 0.05.

## 3. Results

A total of 391 cancer patients participated in the study. Among the participants, 28.4% (*n* = 111) were aged between 39 and 48 years, and approximately 74.4% (*n* = 291) identified as female. The vast majority, 96.2% (*n* = 376), were Saudi citizens. Additionally, more than half of the participants (53.7%, *n* = 210) resided in the Eastern region. Regarding educational attainment, 49.4% (*n* = 193) had bachelor’s degrees. Notably, 28.1% (*n* = 110) of the participants reported having no monthly income.

In summary, the demographic profile reveals that most of the participants are female, aged between 39 and 48, Saudi citizens, living in the Eastern region, educated to a bachelor’s degree level, and receiving no monthly income, as detailed in Table 1.

A bivariate analysis was conducted to examine the relationship between access to healthcare services and various independent variables, including age group, gender, citizenship, region, educational level, income, type of tumor, type of cancer, type of payment, reason for last visit, distance, type of cost, financial support, source of financial support, the impact of virtual appointments on facilitating healthcare services, and acceptance of health applications, as presented in Table 2.

The analysis sought to reveal statistically significant mean differences, as determined by a *t*-test (*t*-value = −1.838, *p*-value < 0.05). Furthermore, an ANOVA test indicated a significant difference between access to healthcare services scores and type of payment (*p*-value < 0.05), suggesting that individuals who pay out of pocket experience reduced access to healthcare services. Additionally, significant differences in access to healthcare services scores were observed for different distances (*p*-value < 0.05), indicating that residents living more than 90 km from healthcare facilities have less access to services.

Moreover, a significant relationship was found between access to healthcare services scores and the acceptance of health applications (*p*-value < 0.05), with those who strongly oppose using such applications demonstrating lower access to healthcare services. In contrast, as summarized in Table 2, no statistically significant differences (*p*-value > 0.05) were identified between access to healthcare services scores and the following variables: age group, gender, citizenship, region, educational level, income, type of cancer, reason for last visit, and financial support.

**Figure 1:**
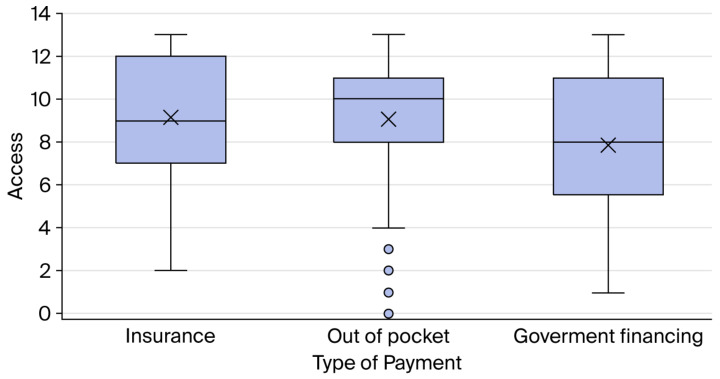
The side-by-side box plots show how the type of payment affects access to healthcare for cancer patients. The “x” symbols stand for the mean, and the circles stand for outliers.

Additionally, a side-by-side box plot illustrates the impact of payment type on access to healthcare services, as shown in Figure 1.

To address the psychometric properties of the composite access score, which aggregates seven binary items (Yes = 1, No = 0; range 0–7, higher scores indicating better access), we examined its underlying structure. Tetrachoric correlations among the items revealed three distinct clusters, suggesting the score reflects multiple domains rather than a single unidimensional construct: a strong digital cluster (e.g., r = 0.919 between virtual appointment preference and perceived helpfulness of virtual appointments), a moderate geographical-financial overlap (e.g., r = 0.691 between missing appointments due to distance and due to cost), and low cross-domain correlations (range: −0.184 to 0.332). Accordingly, three sub-domain scores were derived: geographical (3 items; range 0–3), financial (2 items; range 0–2), and digital (2 items; range 0–2). Inter-domain correlations confirmed their relative independence (geographical–financial: r = 0.37; geographical–digital: r = 0.02; financial–digital: r = −0.04). This multi-domain approach provides a more nuanced, actionable interpretation of access barriers, as detailed in Table 3.

Sub-domain scores were examined against theoretically relevant predictors. Geographical access burden increased markedly with distance, with patients travelling ≥90 km recording the highest mean score (1.98 ± 0.94) compared to those within 10 km (0.91 ± 1.07), a difference that was statistically significant (F = 31.02, *p* < 0.001). Financial access burden was highest among government-financed patients (0.80 ± 0.66) compared to those with insurance (0.49 ± 0.64) or out-of-pocket payment (0.42 ± 0.53; F = 9.02, *p* < 0.001), suggesting that reliance on government financing may reflect greater underlying financial vulnerability rather than better coverage. Digital access scores declined as app acceptance decreased, from a mean of 1.53 (±0.76) among those who strongly agreed to 1.15 (±0.93) among those who strongly disagreed (F = 2.55, *p* = 0.039), indicating that attitudes toward digital health tools are meaningfully associated with digital access engagement.

To assess the robustness of bivariate associations (e.g., payment method and distance) in a multivariable context, an exploratory linear regression was conducted with the composite access score as the outcome and key predictors (payment type [reference: insurance], distance [reference: <10 km], and health application acceptance [reference: strongly agree]) entered simultaneously. The overall model was statistically significant (F(10, 227) = 2.885, *p* = 0.002), explaining 11.3% of the variance (Adjusted R^2^ = 0.074), with no multicollinearity (Variance Inflation Factors (VIF) < 1.3).

Individual effects attenuated somewhat, with out-of-pocket payment (B = −0.463, *p* = 0.077) and shorter distances (e.g., 10–29 km: B = −0.431, *p* = 0.089) approaching significance, while resistance to apps (disagree: B = 0.782, *p* = 0.035) remained independently associated. Note that the analysis used 238 complete cases (39% reduction due to missing distance data via listwise deletion), highlighting interconnected barriers rather than null effects. This strengthens the findings by demonstrating stability while underscoring the multidimensional nature of access.

These findings should be interpreted with caution. The regression analysis was based on 238 complete cases out of 391 (39% reduction due to listwise deletion), primarily driven by missing responses on the distance variable. The attenuation of payment and distance effects in the multivariable model likely reflects the interconnected nature of access barriers—patients facing geographical barriers frequently face financial ones simultaneously—rather than an absence of effect. This pattern underscores the value of examining access as a multidimensional construct, as reported in the sub-domain analysis above.

## 4. Discussion

The study assessed the factors associated with access to healthcare services for cancer patients in Saudi Arabia, utilizing a health belief model, and the findings indicate that cancer patients face numerous challenges in accessing healthcare services. The main challenges highlighted include the type of payment, distance to healthcare services, type of tumor, and acceptance of healthcare applications. These factors significantly impact cancer patients’ access to healthcare services. Conversely, no statistically significant differences were found between access to healthcare services scores and variables such as age group, gender, citizenship, region, educational level, income, cost, type of cancer, reason for last visit, and financial support.

The findings of this study can be meaningfully interpreted through the lens of the Health Belief Model (HBM) [25,26,27,28], which posits that health behaviors, including healthcare utilization, are driven by perceived susceptibility (risk awareness), severity (consequences of inaction), benefits (advantages of action), barriers (obstacles to action), cues to action (triggers for behavior), and self-efficacy (confidence in performing the behavior). In this context, our results explicitly map to these constructs, providing a structured explanation for access patterns among Saudi cancer patients.

For instance, significant associations with payment method and distance align with the HBM’s “perceived barriers” construct, where financial burdens (e.g., out-of-pocket payments, highest in government-financed patients per sub-domain analysis) and logistical hurdles (e.g., ≥90 km travel, linked to elevated geographical burden) deter timely care-seeking, potentially exacerbating disease progression in malignant tumor cases (t = −1.838, *p* = 0.037) [27,28]. Tumor type further reinforces perceived severity, as malignant cases demand more frequent interactions, amplifying these barriers. Conversely, acceptance of health applications and virtual appointments reflects “perceived benefits” and “self-efficacy,” with strong agreement linked to higher digital sub-domain scores (F = 2.55, *p* = 0.039) and overall access (F = 113.633, *p* < 0.001), suggesting that digital tools boost confidence in overcoming structural obstacles [26]. These may serve as “cues to action,” especially under Vision 2030’s digital transformation push [24]. The multivariable attenuation of payment and distance effects (Table 4) highlights how barriers interconnect, reducing self-efficacy when multiple HBM elements compound—yet digital resistance independently predicts lower access (*p* = 0.035), underscoring modifiable perceptual factors. Overall, the HBM elucidates why structural barriers dominate in resource-constrained settings like Saudi Arabia, while digital readiness enhances benefits and efficacy to mitigate them.

Regarding the factors influencing access to healthcare services, the analysis revealed that the type of payment significantly affects healthcare access, with individuals who pay out of pocket experiencing reduced access to services. Previous studies validate this conclusion, indicating that payment methods influence healthcare accessibility. The variability in payment options can be attributed to the Kingdom of Saudi Arabia’s current financial structure, which predominantly relies on government funding and results in fewer individuals having health insurance compared to other countries. Concerns regarding health insurance access are notably affected by the privatization of hospitals. As a result, findings indicate that only a small fraction of payments are made through insurance, with government funding accounting for only 11.5% of total service costs, while out-of-pocket payments constitute 75.4% [32,33,34,35,36]. Other studies have suggested that health insurance facilitates access to care by mitigating the high and often unexpected costs of medical treatment and by linking patients to healthcare provider networks. While the results of our study are consistent over time, they may change if privatization occurs in Saudi Arabia, potentially leading to increased health insurance uptake, which could alter the payment landscape for healthcare services [37,38,39].

Additionally, this study identified significant differences in access to healthcare services scores based on distance, indicating that individuals residing more than 90 km from healthcare facilities have diminished access. Several studies confirm that distance is a barrier to accessing healthcare services, with increased distance from a healthcare institution correlating with decreased access [4,13,14]. The frequency of clinic visits and overall cancer management are influenced by distance, as the incapacitating nature of cancer necessitates regular appointments for screening and monitoring. Travel challenges can impede timely follow-up care, highlighting the need for innovative solutions such as online virtual appointments to enhance accessibility. While advancements in cancer management continue, significant disparities in access to high-quality cancer care persist globally, leaving a considerable portion of the population without basic health care services.

Differences in access to healthcare services were also related to tumor type, with malignant tumors presenting more significant challenges than benign ones. Delays in diagnosis and treatment due to long waiting times in the public health system contribute to disease progression for patients with malignant tumors [40].

Moreover, acceptance and willingness to use health applications significantly influenced access to healthcare services. This finding aligns with our second research objective, which aimed to propose alternative solutions to enhance access. A positive reception for health applications among cancer patients indicates a strong need for remote follow-up and consultations. Consequently, we recommend developing a dedicated health application for monitoring cancer cases and providing remote consultations. Research shows that new regulations regarding the meaningful use of Electronic Health Records (EHRs) [41,42,43,44].

Conceptually, while this study emphasizes structural dimensions of access (e.g., distance, payment, digital tools) as per the HBM’s barrier focus, access is widely recognized as multidimensional, extending beyond binary availability to include approachability, acceptability, appropriateness, responsiveness, coordination, and continuity of care [11,18]. In Saudi Arabia’s context, structural barriers may disproportionately affect initial approachability (e.g., distance) and affordability (e.g., payment), but our findings suggest gaps in coordination (e.g., virtual tools as bridges) and continuity (e.g., follow-up challenges for malignant tumors). For instance, government-financed patients’ higher financial burden (Table 3) may signal coordination issues in navigating public systems, while digital acceptance could enhance responsiveness by enabling timely consultations. Integrating these dimensions strengthens the interpretation: even with Vision 2030’s reforms, unaddressed non-structural elements like cultural acceptability of virtual care or inter-provider coordination could perpetuate inequities. Future research should operationalize these (e.g., via validated multi-domain scales) to evaluate holistic interventions, such as patient navigation programs tailored to HBM constructs [20,21]. This would build on our structural insights without deferring the conceptual depth.

The study also found significant differences pertaining to care coordination, suggesting that Health Information Technology (HIT) infrastructure is essential in terms of enhancing continuity of care between cancer specialists and patients, as well as among different healthcare providers [45].

In contrast to prior studies, our results indicated no statistical significance in access to healthcare services based on age group or gender. Previous research has often highlighted significant disparities in access related to these factors, suggesting that some countries exhibit gender biases, with women receiving fewer health services due to decision-making constraints and prioritization of men [46]. Other authors have argued that while gender may not be a barrier, age can be significant, with younger individuals demonstrating greater demand for health services regardless of gender [29,47].

Furthermore, our findings suggest no significant differences in access to healthcare based on educational level. However, a study conducted in rural Gambia concluded that education significantly influences access to healthcare, with individuals in lower educational brackets more likely to resort to traditional medicine, while those with higher education experience more frequent health check-ups. This suggests that higher educational attainment positively impacts access to health services [30].

Regarding healthcare utilization, a study comparing unauthorized and authorized immigrants to U.S.-born individuals found no significant differences in healthcare expenditure between these groups, although both immigrant populations did exhibit lower expenditure than their U.S.-born counterparts [48,49]. Similarly, geographical variations in healthcare access have been observed in studies focusing on attention deficit hyperactivity disorder (ADHD), where no significant associations were found despite tax-funded access to healthcare [50]. Additionally, a study in Shenzhen, China, examining the health needs of insured and uninsured migrant workers, concluded that no significant income differences were observed between the two groups [35].

In terms of cancer type, our findings indicate that the type of cancer does not significantly impact access to healthcare facilities for patients in Saudi Arabia. This contrasts with studies from the United States, the United Kingdom, and Canada, which consistently demonstrate a relationship between cancer type and patient experiences, suggesting that positive experiences correlate with better access to healthcare services [51]. The limitations of existing studies examining this relationship highlight the need for further research, as access disparities related to cancer type remain underexplored [17].

Lastly, our study revealed no correlation between access to healthcare services and the reason for a patient’s last visit to a healthcare facility. This finding is unexpected, particularly given the significant association between financial support and access to healthcare services in other studies. We anticipated a positive correlation; however, our results did not support this [1,8,48,52]. A study conducted in a teaching hospital in Riyadh, Saudi Arabia, emphasized the necessity for the Saudi Ministry of Health and other healthcare providers to recognize the financial burdens accompanying any cancer diagnosis and to implement support measures to ensure that travel, childcare, and other out-of-pocket costs do not become barriers to accessing essential healthcare services [31].

Nevertheless, this study has several limitations. Firstly, the vast majority of the sample was drawn from the Eastern Province of Saudi Arabia, which may limit the generalizability of the findings to other regions. Secondly, utilizing the online survey that was distributed via social media might underrepresent older, less literate, lower-income, and rural populations. Thirdly, the sensitivity of cancer patients regarding disclosing information about their condition posed data collection challenges, resulting in non-responses and an extended data collection period. Lastly, the regression used listwise deletion, which reduced the sample from 391 to 238 (39% loss), primarily due to missing responses on the distance variable. This limits the generalizability of the multivariable findings and should be noted explicitly as a limitation. However, the study’s strengths include its novelty, as it is the first study of its kind conducted in Saudi Arabia. Additionally, the sample was rigorously defined, ensuring that only participants within the specified criteria were included in the study.

## 5. Conclusions

This study identified several factors influencing access to healthcare services for cancer patients, including payment method, distance, type of cancer, and acceptance of health applications. These factors were found to significantly impact access to healthcare services. In fact, patients travelling ≥90 km had the highest geographical burden while government-financed patients carried the highest financial burden. Through the HBM lens, these reflect key perceived barriers and benefits, with digital tools offering self-efficacy boosts. Based on the health belief model, the analysis revealed no statistically significant differences in access scores according to age group, gender, citizenship, region, educational level, income, type of cancer, reason for the last visit, or financial support. Recognizing access as multidimensional—encompassing structural, perceptual, and process-oriented elements—highlights opportunities for integrated solutions. Furthermore, digital access decreased as app acceptance declined. Therefore, fostering cancer patients’ acceptance of health applications can enhance the utilization of remote appointments and consultations, thereby improving access to necessary healthcare services.

## Figures and Tables

**Table 1:** Descriptive analysis of demographic data.

Variables	Frequency (%)
Age	
- 18–28	93 (23.8)
- 29–38	88 (22.5)
- 39–48	111 (28.4)
- 49–58	71 (18.2)
- 59 and above	28 (7.2)
Gender	
- Male	100 (25.6)
- Female	291 (74.4)
Citizenship	
- Citizen	376 (96.2)
- Resident	15 (3.8)
Region	
- Northern	21 (5.4)
- Eastern	210 (53.7)
- Central	88 (22.5)
- Western	49 (12.5)
- Southern	23 (5.9)
Educational level	
- No formal education	10 (2.6)
- Elementary education	8 (2.0)
- Intermediate education	25 (6.4)
- Secondary education	100 (25.6)
- Diploma	41 (10.5)
- Bachelor’s degree	193 (49.4)
- Postgraduate degree	14 (3.6)
Monthly income	
- Below 3000	61 (15.6)
- 3000 to 5999	34 (8.7)
- 6000 to 8999	30 (7.7)
- 9000 to 11,999	36 (9.2)
- 12,000 to 14,999	29 (7.4)
- 15,000 and above	43 (11.0)
- No monthly income	110 (28.1)
- Prefer not to say	48 (12.2)

**Table 2:** Bivariate analysis.

Variables	Access to Healthcare	Test (*p*-Value)
	Mean (SD)	
Age		
- 18–28	9.0 (2.6)	*f* = 0.266 (0.900)
- 29–38	8.8(3.0)	
- 39–48	8.8 (2.8)	
- 49–58	9.2 (2.7)	
- 59 and above	9.1 (2.7)	
Gender		
- Male	8.7 (3.1)	*t* = −1.236 (0.130)
- Female	9.1 (2.7)	
Citizenship		
- Citizen	9.0 (2.8)	*t* = 2.118 (0.811)
- Resident	7.5 (2.6)	
Region		
- Northern	9.2 (2.5)	*f* = 2.085 (0.082)
- Eastern	8.7 (2.9)	
- Central	9.6 (2.5)	
- Western	9.2 (2.6)	
- Southern	8.3 (3.1)	
Educational level		
- No formal education	10.6 (2.8)	*f* = 1.302 (0.255)
- Elementary education	7.5 (2.0)	
- Intermediate education	9.1 (2.9)	
- Secondary education	9.2 (2.6)	
- Diploma	8.8 (3.0)	
- Bachelor’s degree	8.9 (2.8)	
- Postgraduate degree	8.3 (2.8)	
Monthly income		
- Below 3000	8.7 (2.6)	*f* = 0.744 (0.635)
- 3000 to 5999	8.7 (3.1)	
- 6000 to 8999	9.7 (2.4)	
- 9000 to 11,999	8.9 (2.6)	
- 12,000 to 14,999	8.8 (2.9)	
- 15,000 and above	9.1 (2.9)	
- No monthly income	8.7 (2.9)	
- Prefer not to say	9.4 (2.5)	
Type of tumor		
- Benign	8.4 (3.2)	*t* = −1.838 (0.037)
- Malignant	9.1 (2.7)	
Type of payment		
- Insurance	9.1 (2.7)	*f* = 3.782 (0.024)
- Out of pocket	9.1 (2.7)	
- Government financing	7.9 (3.1)	
Reason for last visit		
- Chemotherapy	8.7 (2.8)	*f* = 0.945 (0.438)
- Surgery	9.2 (2.7)	
- Radiation Therapy	8.2 (2.7)	
- Follow-up	9.1 (2.8)	
- Other	9.3 (2.3)	
Type of cancer		
- Breast cancer	9.3 (2.7)	*f* = 1.201 (0.308)
- Lung cancer	8.7 (3.3)	
- Prostate cancer	9.2 (3.2)	
- Stomach cancer	8.4 (2.5)	
- Skin cancer	7.7 (4.0)	
- Other	8.8 (2.7)	
Distance		
- Less than 10 km	10.0 (2.2)	*f* = 10.534 (0.000)
- 10–29 km	10.1 (2.2)	
- 30–49 km	8.8 (2.10)	
- 50–69 km	8.4 (3.0)	
- 70–89 km	9.3 (1.9)	
- 90 km and more	7.9 (3.0)	
Type of cost		
- Transportation	6.0 (3.5)	*f* = 0.794 (0.501)
- Treatment	6.5 (2.5)	
- Housing	6.9 (2.6)	
- Other	4.7 (1.2)	
Financial support		
- Yes	9.0 (2.6)	*t* = 0.112 (0.202)
- No	9.0 (2.9)	
Type of financial support		
- Government	9.1 (2.6)	*f* = 0.957 (0.416)
- Relatives	9.0 (1.8)	
- Friends	6.0 (0.0)	
- Charity	8.5 (3.9)	
Do virtual appointments contribute to facilitating access to healthcare services?		
- Yes	9.1 (2.7)	*t* = 2.175 (0.215)
- No	8.4 (3.0)	
Acceptance rate of using health applications to get an appointment		
- Strongly agree	11.0 (1.7)	*f* = 113.633 (0.000)
- Agree	9.2 (1.9)	
- Neutral	7.4 (2.1)	
- Disagree	5.6 (2.1)	
- Strongly disagree	3.7 (2.6)	

**Table 3:** Differences in sub-domain scores across relevant predictors.

Sub-Domain	Predictor	Group	*n*	Mean (SD)	F	*p*
Geographical	Distance	<10 km	54	0.91 (1.07)	31.02	<0.001
		10–29 km	95	0.58 (0.89)		
		30–49 km	44	0.70 (0.98)		
		50–69 km	27	1.04 (1.16)		
		70–89 km	21	1.05 (0.80)		
		≥90 km	150	1.98 (0.94)		
Financial	Payment type	Insurance	51	0.49 (0.64)	9.02	<0.001
		Out of pocket	295	0.42 (0.53)		
		Government	45	0.80 (0.66)		
Digital	App acceptance	Strongly agree	150	1.53 (0.76)	2.55	0.039
		Agree	108	1.56 (0.69)		
		Neutral	84	1.29 (0.90)		
		Disagree	25	1.40 (0.87)		
		Strongly disagree	20	1.15 (0.93)		

**Table 4:** Multivariate Regression.

Predictor	B	SE	95% CI	*p*
(Intercept)	2.973	0.304	[2.374, 3.571]	<0.001
Payment: Government	0.454	0.388	[−0.310, 1.217]	0.243
Payment: Out of pocket	−0.463	0.261	[−0.977, 0.051]	0.077
Distance: 10–29 km	−0.431	0.252	[−0.927, 0.066]	0.089
Distance: 30–49 km	−0.057	0.301	[−0.650, 0.537]	0.850
Distance: 50–69 km	0.334	0.352	[−0.359, 1.027]	0.343
Distance: 70–89 km	0.115	0.387	[−0.648, 0.877]	0.767
App: Agree	0.194	0.229	[−0.258, 0.647]	0.398
App: Neutral	−0.116	0.264	[−0.635, 0.404]	0.661
App: Disagree	0.782	0.368	[0.057, 1.507]	0.035
App: Strongly disagree	−0.239	0.492	[−1.208, 0.730]	0.628

## Data Availability

The data are not publicly available due to privacy and ethical restrictions pertaining to cancer patients in Saudi Arabia.

## Appendix A. The Data Collection Instrument

We are students of Health Information Management and Technology, College of Public Health at Imam Abdulrahman bin Faisal University. We invite you to participate in this survey by completing the questionnaire for our graduation project titled: “Factors Associated with Access to Healthcare Services for Cancer Patients in Saudi Arabia.” The aim of this study is to identify barriers affecting access to healthcare services for cancer patients and to suggest potential solutions to improve access. The questionnaire will take approximately 5 min to complete. Participation is voluntary, and all responses will remain confidential and anonymous, with no identifiable personal information collected or shared with unauthorized parties. Thank you for your time and cooperation.

For inquiries: 2200000498@iau.edu.sa.

**Section (1) demographic characteristics:** PLEASE ANSWER THE FOLLOWING QUESTIONS: 1- Age: - 18–28 - 29–38 - 39–48 - 49–58 - 59 and above 2- Gender: - Male - Female 3- Citizenship: - Citizen - Resident 4- Region: - Northern - Eastern - Central - Western - Southern 5- Educational level: - No formal education - Elementary education - Intermediate education - Secondary education - Diploma’s degree - Bachelor’s degree - Postgraduate degree 6- The amount of monthly income (approximately): - Below 3000 - 3000 to 5999 - 6000 to 8999 - 9000 to 11,999 - 12,000 to 14,999 - 15,000 and above - No monthly income - Prefer not to say
**Section (2) frequent questions:** 1- In order to answer the following questions, please specify whether you are the patient or the accompanying person: - The patient - Accompanying person * If you are the accompanying person, what is your relationship with the patient? - Mother - Father - Wife - Husband - Son - Daughter - Sister - Brother - Other: 2- What type of tumor do you have? - Benign - Malignant 3- Which type of payment you use to have the health care services? - Insurance - Out of pocket - Government financing 4- What was the reason for your last visit? - Chemotherapy - Surgery - Radiation Therapy - Follow-up - Other 5- What type of cancer have you been diagnosed with? - Breast cancer - Lung cancer - Prostate cancer - Stomach cancer - Skin cancer - Other 6- Are the healthcare facilities available in your city of residence? - Yes - No 7- How do you rate your access to healthcare facilities? - Very satisfied - Satisfied - Neutral - Dissatisfied - Very dissatisfied 8-How far is the healthcare facility where did you receive your care, from your city of residence? - less than 10 km - 10–29 km - 30–49 km - 50–69 km - 70–89 km - 90 km and more 9- Have you ever missed an appointment because of the distance between your home and the healthcare facility? - Yes - No 10- Have you ever had to navigate from one city to another within Kingdom to receive healthcare services? - Yes - No If the answer is yes, did you have to live in that area? - Yes - No 11- Do you feel that the healthcare provider’s language affect access to get appropriate healthcare services? - Yes - No 12- If you face other difficulties in accessing healthcare services, mention them here 13- Have you ever missed an appointment because of the cost? - Yes - No If the answer is yes, what is the type of cost? - For transportation - For treatment - For housing - Other 14- Do you receive financial support to get healthcare services? - Yes - No 15- If the answer is yes, what are the sources of financial support that you receive to get healthcare services? - The government - Relatives - Friends - Charity 16- As a cancer patient, would you prefer to have virtual appointments? - Yes - No 17- Do you think that virtual appointments contribute to facilitating access to healthcare services? - Yes - No 18- How would you rate your acceptance to use health applications to get an appointment? - Strongly agree - Agree - Neutral - Disagree - Strongly disagree
**Sources:** [1,5,6,10,13,15,17,29,30,31,53].
